# A Systematic Review and Meta-Analysis of Penis Length and Circumference According to WHO Regions: Who has the Biggest One?

**DOI:** 10.5152/tud.2025.24038

**Published:** 2025-03-06

**Authors:** Hadi Mostafaei, Keiichiro Mori, Satoshi Katayama, Fahad Quhal, Benjamin Pradere, Takafumi Yanagisawa, Ekaterina Laukhtina, Frederik König, Reza Sari Motlagh, Pawel Rajwa, Hanieh Salehi-Pourmehr, Sakineh Hajebrahimi, Shahrokh F. Shariat

**Affiliations:** 1 Department of Urology, Comprehensive Cancer Center, Medical University of Vienna, Vienna, Austria; 2 Research Center for Evidence-Based Medicine, Iranian EBM Center: A JBI Centre of Excellence, Tabriz University of Medical Sciences Faculty of Medicine, Tabriz, Iran; 3 Department of Urology, The Jikei University School of Medicine, Tokyo, Japan; 4 Department of Urology Okayama University Graduate School of Medicine, Dentistry and Pharmaceutical Sciences, Okayama, Japan; 5 Department of Urology, King Fahad Specialist Hospital-Dammam, Saudi Arabia; 6 Institute for Urology and Reproductive Health, Sechenov University, Moscow, Russia; 7 Department of Urology, University Medical Center Hamburg-Eppendorf, Hamburg, Germany; 8 Men’s Health and Reproductive Health Research Center, Shahid Beheshti University of Medical Sciences, Tehran, Iran; 9 Department of Urology, Medical University of Silesia, Zabrze, Poland; 10 Medical Philosophy and History Research Center, Tabriz University of Medical Sciences, Tabriz, Iran; 11 Department of Urology, University of Texas Southwestern Medical Center, Dallas, TX, USA; 12 Department of Urology, Charles University Second Faculty of Medicine, Prague, Czech Republic; 13 Department of Urology, Weill Cornell Medical College, New York, New York, USA; 14 Karl Landsteiner Institute of Urology and Andrology, Vienna, Austria; 15 Hourani Center for Applied Scientific Research, Al-Ahliyya Amman University, Amman, Jordan

**Keywords:** Penis, length, circumference, world health organization

## Abstract

This study aimed to perform a systematic review and meta-analysis of stretched, erect, and flaccid penis length as well as circumference according to geographic WHO regions. PubMed, Embase, Scopus, and Cochrane Library were searched for articles published until February 2024. Studies in which a healthcare professional evaluated the penis size were considered eligible. After assessing the risk of bias, a systematic review and meta-analyses were performed according to the Preferred Reporting Items for Systematic Review and Meta-analysis statement, and the outcomes were grouped based on the WHO regions. A total of 33 studies comprising 36 883 patients were included. The risk of bias in the included studies was moderate/low. A comprehensive systematic review was done and meta-analyses performed for flaccid length [n = 28 201, mean (SE) 9.22 (0.24) cm], stretched length [n = 20 814, mean (SE) 12.84 (0.32) cm], erect length [n = 5669, mean (SE) 13.84 (0.94) cm], flaccid circumference [n = 30 117, mean (SE) 9.10 (0.12) cm], and erect circumference [n = 5168, mean (SE) 11.91 (0.18) cm]. The mean length of the stretched penis was largest in Americans [14.47 (0.90) cm]. The mean length of the flaccid penis was the largest in the Americas [10.98 (0.064) cm]. The mean flaccid penile circumference was largest in Americans [n = 29 714, mean (SE) 10.00 (0.04) cm]. Penis sizes vary across WHO regions, suggesting the need to adjust standards according to geography to better understand councilmen and their partners. These data provide a framework for discussing body image expectations and therapeutic strategies in this sensitive and emotional subject matter.

## Introduction

What is the normal penis size? This has been a longstanding topic of interest.^1,2^ Due to cultural and social backgrounds, the size of the penis has been linked with masculinity, virility, and sexual power in men.^3^ Misleading information in the media as well as pornography have led to anxiety and a sense of inadequacy in men underlined by the belief that the size of the penis is directly correlated with sexual satisfaction.^2,4^ Not surprisingly, a growing number of men believe that their penises are smaller than the average penis size.^5^ In a survey of 52 031 heterosexual men and women regarding views on penis size, 55% of men were not fulfilled with their penis size, but 85% of women were satisfied with their partner’s penis size.^1^ This perception may lead to body image disorders ranging the spectrum from small penis anxiety (SPA)^6^ to penile dysmorphic disorder (PDD) (i.e. preoccupation with repeated behaviors lasting more than one hour per day).^6,7^ Attempts to better understand the normative range of penis size have been inconsistent in different measurement approaches.^8,9^ The study by Veale and colleagues, published in 2015, provided solid information about the normal distribution of penis size for men in general.^8^ However, recent data support the long-held assumption that penile sizes vary according to geographical regions.^10^

Due to measurement limitations in evaluating erect penis size, the main purpose of the present study is to perform a systematic review and meta-analysis to assess the stretched and flaccid penis lengths as well as flaccid penis circumference in men according to the WHO geographic regions.

## Materials and Methods

A protocol was registered in the International prospective register of systematic reviews (PROSPERO) Identification number: CRD42021251431. We followed the PRISMA (preferred reporting items for systematic reviews and meta-analyses) 2020 guidelines.

Inclusion/Exclusion Criteria

### Searching Databases

**Figure 1.**
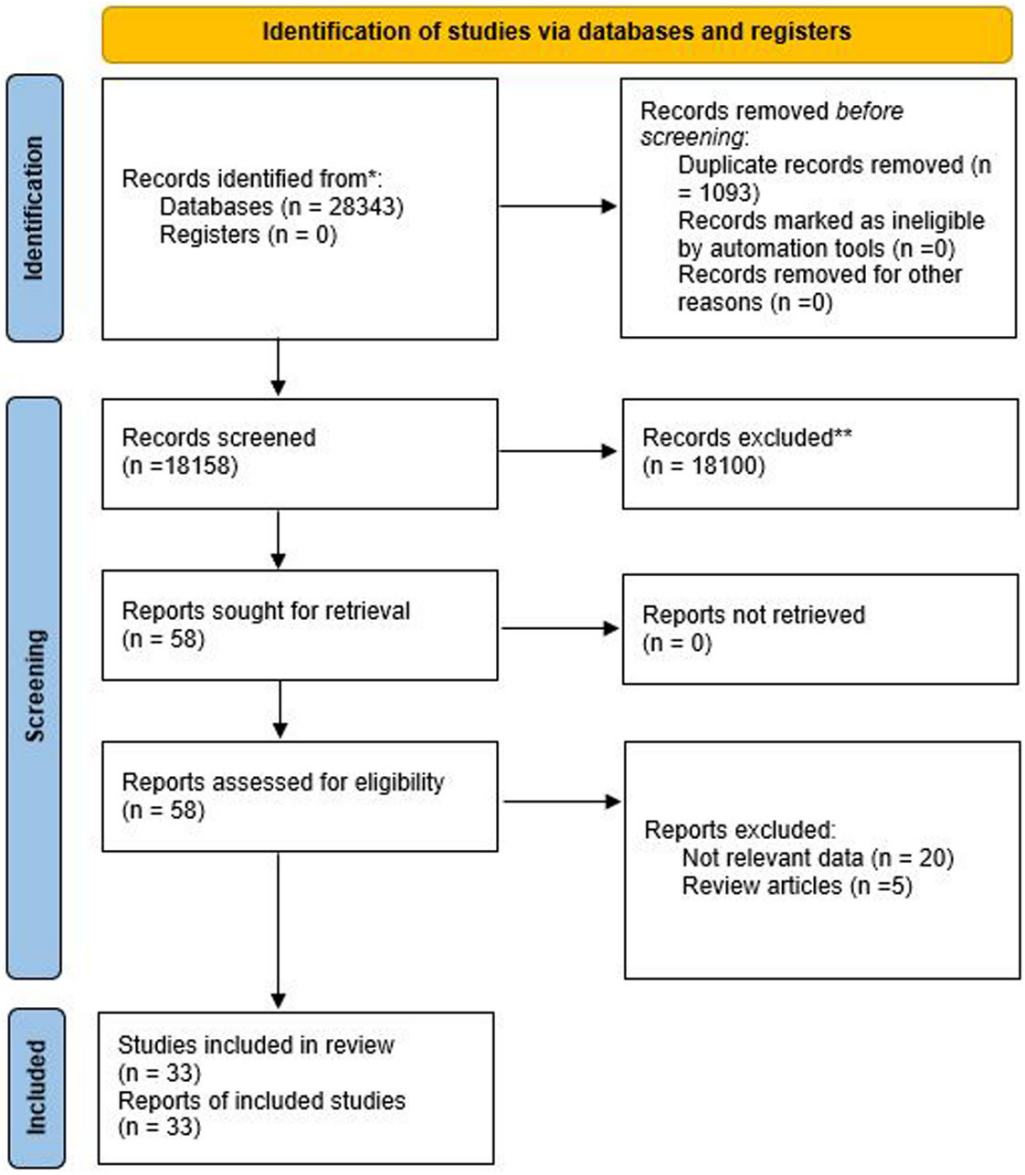
PRISMA flow diagram. Abbreviation: PRISMA, Preferred reporting items for systematic reviews and meta-analyses.

PubMed, Embase, Scopus, and Cochrane Library were searched for articles published until February 2024. We used original and Mesh keywords including the penis, phallus, genital, organ size, organ volume, and organ girth to search the related documents. Figure 1 represents the flow diagram of screening-related articles. The following search strategy was applied in the PubMed database:

((“Penis”[Mesh]) OR (Penis [Text Word] OR penile [Text Word] OR phallus [Text Word])) AND ((“Organ Size”[Mesh]) OR (Size [Text Word] OR girth [Text Word] OR measurement [Text Word] OR length [Text Word] OR circumference [Text Word] OR dimension*[Text Word])). The search strategy of databases is available in Appendix 1.

### Inclusion and Exclusion Criteria

We included any study which evaluated penis size in different regions of the world. Studies in which a healthcare professional evaluated the penis size were considered eligible. All observational studies including prospective and retrospective cohort, case-control, and cross-sectional studies were included in this review. Studies published in English from inception till February 2024 were considered for inclusion in the current review.

Self-reported evaluations, patients with a history of certain urologic ailments, complaints of penis size and erectile/sexual dysfunction, history of genital reconstructive surgery as well as congenital or acquired genital anomalies were excluded.

The primary and secondary outcomes clinically measurement of stretched and flaccid penis lengths, and flaccid penis circumference.

However, information about how these measures were standardized across studies is needed.

For categorization the world regions, we used World Health Organization (WHO) regions. The WHO regions include the African Region (AFRO), the Region of the Americas (AMRO), the Eastern Mediterranean Region (EMRO), the European Region (EURO), the South-East Asia Region (SEARO), and the Western Pacific Region (WPRO) https://www.who.int/countries.

### Study Selection

Following the search, all identified citations were imported into EndNote X9.1 and duplicates were removed. Two independent reviewers screened titles and abstracts based on the eligibility criteria of the review. The full text of potentially eligible studies was retrieved and assessed in detail against the inclusion criteria by the mentioned reviewers. Studies that did not meet the inclusion criteria were excluded. Any reviewer disagreements were resolved through discussion, or by referring to a third reviewer.

### Assessment of Methodological Quality

Two independent reviewers critically appraised eligible studies at the study level using standardized critical appraisal instruments from the Joanna Briggs Institute JBI Critical Appraisal Checklist (https://jbi.global/critical-appraisal-tools) for Analytical Cross-Sectional, Observational Cohort and/or Case-Control Studies. Any disagreements between the reviewers were resolved through discussion. The details of the study qualities are presented in Table 1.

### Data Extraction

Two independent reviewers extracted data from included studies using a modified standardized JBI data extraction tool. The data extracted included populations, sample size, study methods, publication year, region of study, and outcome measurement. The reviewers resolved disagreements through discussion.

### Data Synthesis

The comprehensive meta-analysis tool v3.7z was used to conduct the meta-analysis. The Q statistic was used to identify heterogeneity within the studies. Additionally, the I^2^ statistic was used to calculate the effect of study heterogeneity. Low I^2^ was defined as 25%, moderate as 25%-75%, and high as >75%. A fixed-effect model was used when there was no statistically significant difference in the heterogeneity (*P *< .05); otherwise, a random-effect model was applied.

## Results

The PRISMA diagram shows the number of articles that are included (Figure 1). A total of 33 studies comprising 36 883 patients were included. The risk of bias in the included studies was moderate/low; thus, no study was excluded.

A comprehensive systematic review and meta-analyses were performed. In 5669 men, the mean erect penile length was 13.84 cm (SE = 0.94). The mean length of a flaccid penis in 28 201 men was 9.22 cm (SE = 0.24) and the mean stretched penile length in 20 814 men was 12.84 cm (SE = 0.32). The mean flaccid circumference, in 30 117 men, was 9.10 cm (0.12) and the mean erect circumference, in 5168 men, was 11.91 cm (0.18).

**Figure 2.**
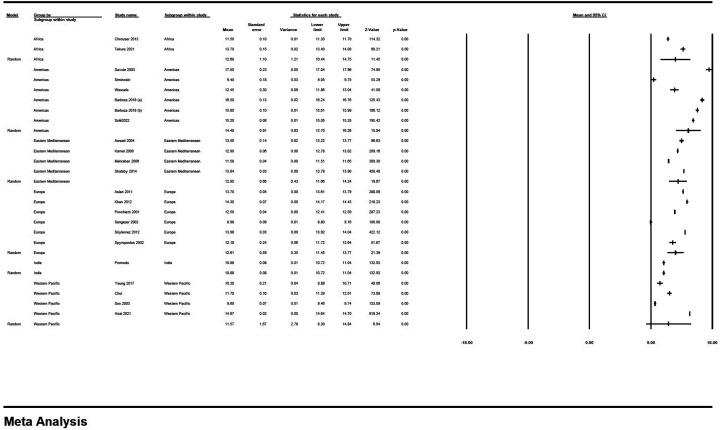
The mean length of stretched penis in different WHO regions. Abbreviation: WHO, World Health Organization.

**Figure 3.**
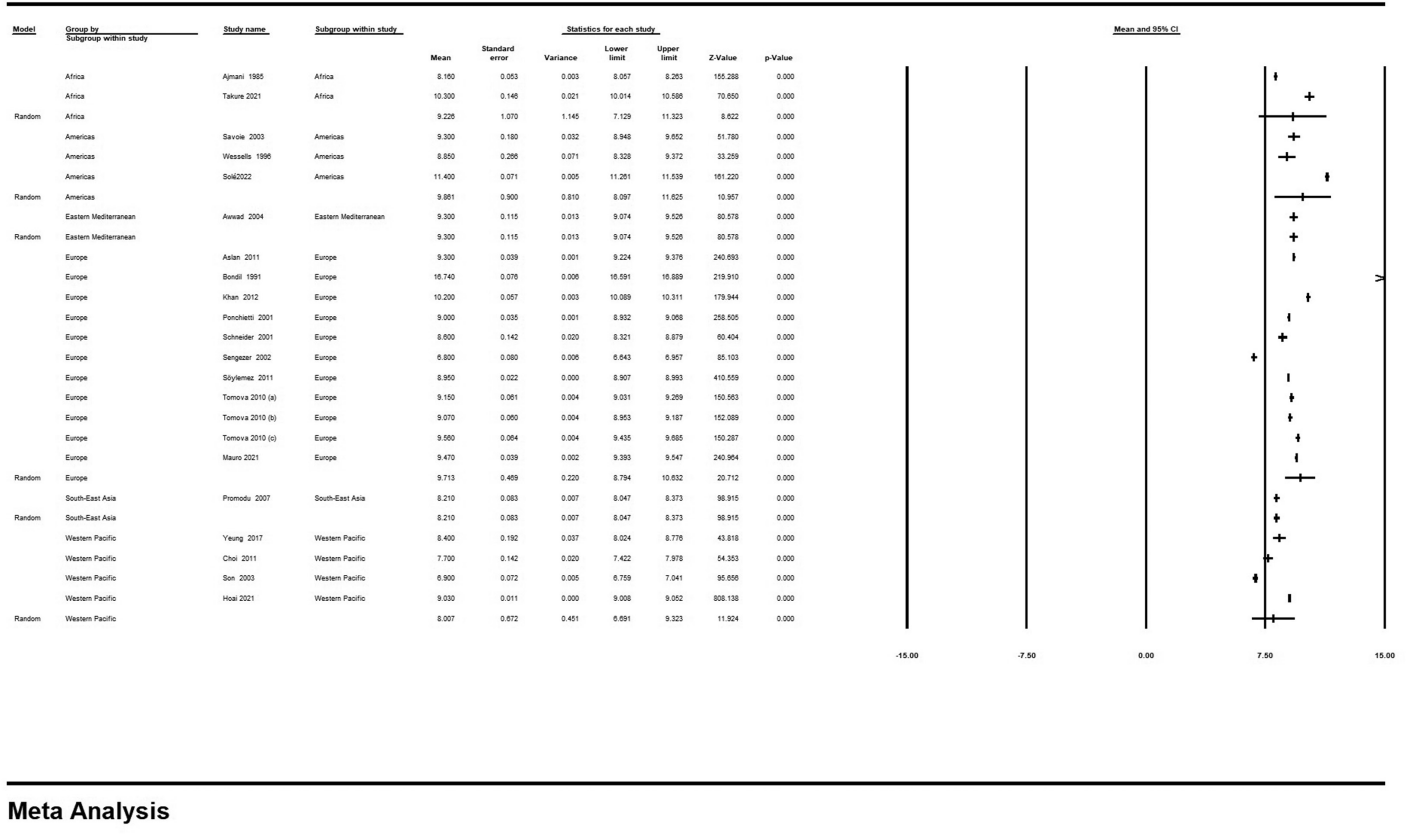
The mean length of flaccid penis in different WHO regions. Abbreviation: WHO, World Health Organization.

**Figure 4.**
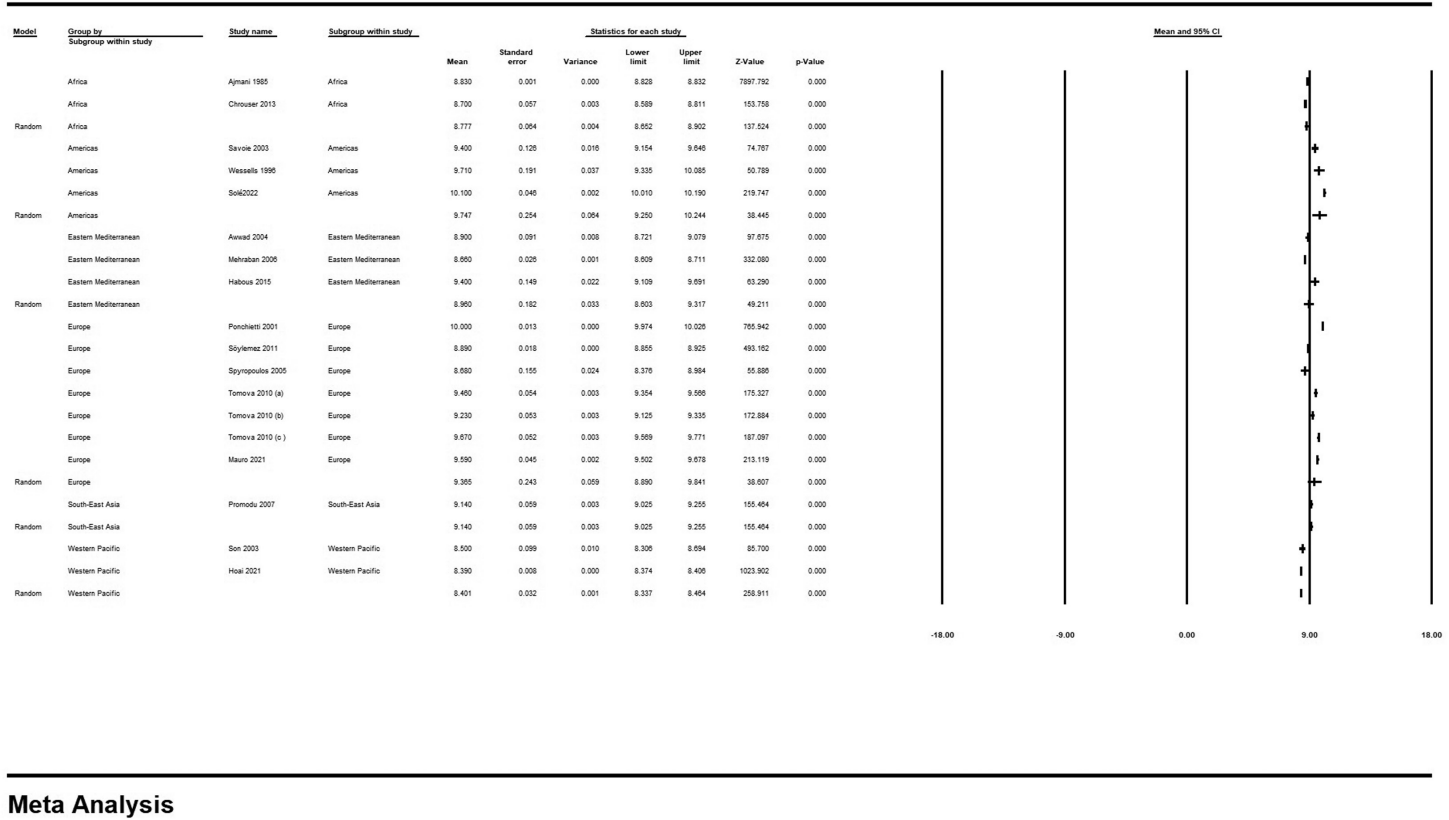
The mean flaccid penile circumference in different WHO regions. Abbreviation: WHO, World Health Organization.

**Figure 5.**
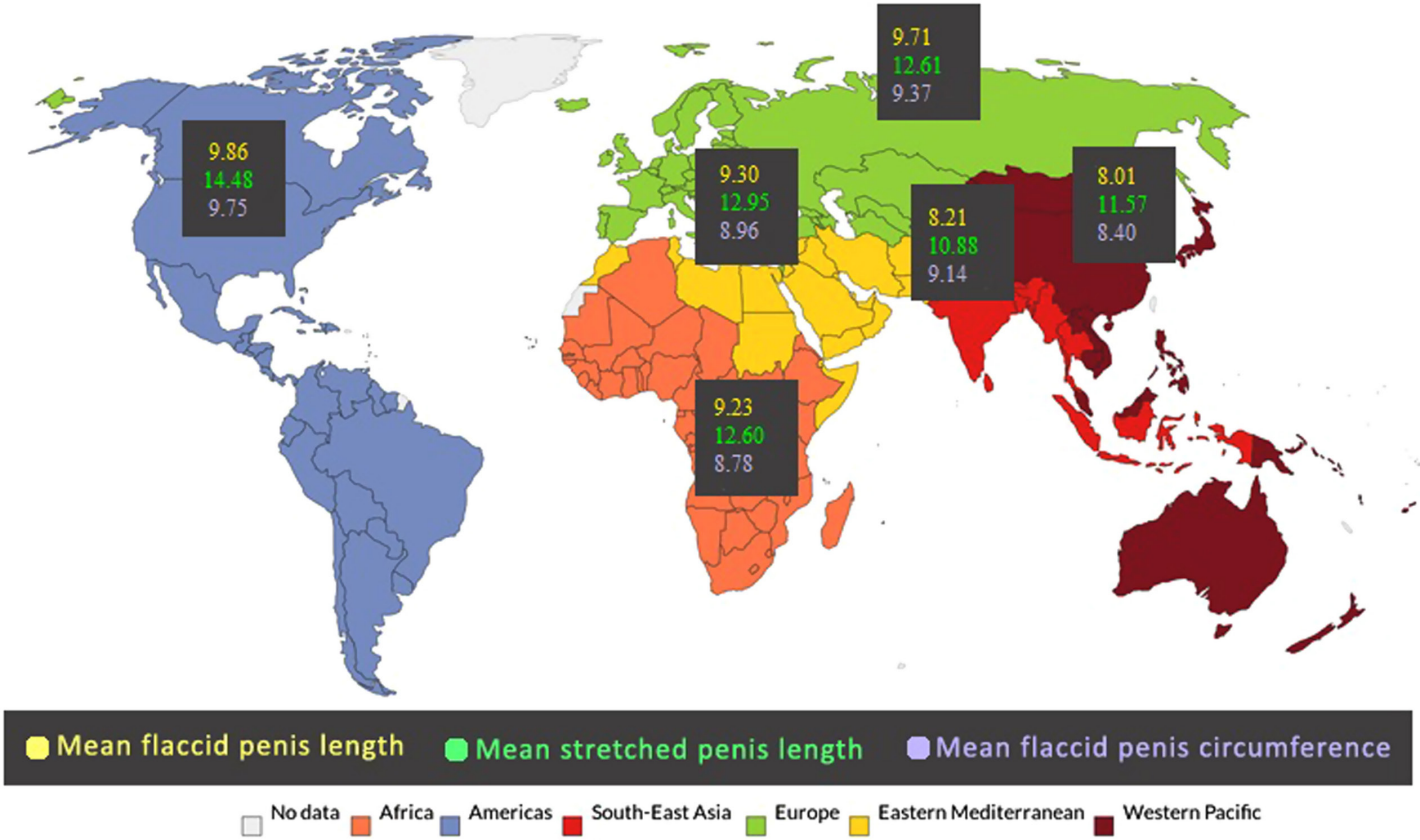
The world map of the size of the penis.

**Figure 6.**
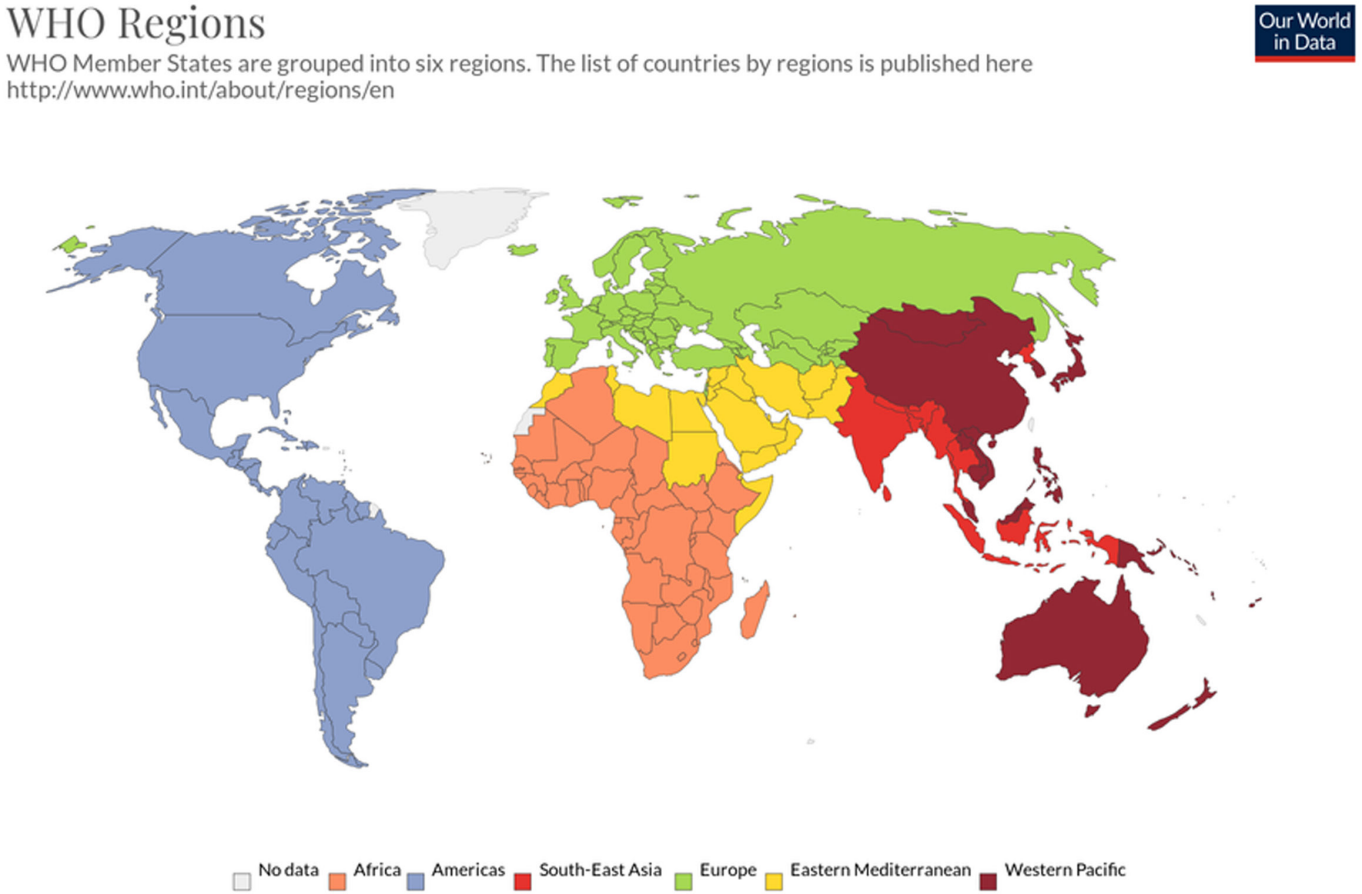
WHO regions. Abbreviation: WHO, World Health Organization.

In the subgroup analyses of penile length and circumference based on the WHO world regions, we found that the mean length of stretched penis was largest in men living in the Americas [14.47 (0.90) cm] followed, by decreasing order, by those living in Eastern Mediterranean [12.95 (0.66) cm], Europeans [12.61 (0.59) cm], Africans [12.59 (1.1) cm South-East Asians [10.88 (0.08) cm] and Western Pacific men [11.57 (1.66) cm] (Figure 2). The mean length of flaccid penis was largest in Americans [9.86 (0.90), followed by Europeans [9.71 (0.46) cm], East Mediterranean [9.30 (0.12) cm], Africans [9.22 (1.07) cm], South-East Asians [8.21 (0.08) cm], and Western Pacific men [8.00 (0.67) cm] (Figure 3). The mean flaccid penile circumference was largest in Americans [9.74 (0.25) cm] followed by Europeans [9.36 (0.24) cm], South-East Asians [9.14 (0.06) cm], and Eastern Mediterranean [8. 96 (0.18) cm], Africans [8.78 (0.06) cm] and Western Pacific men [8.40 (0.03) cm]. (Figure 4). Our study shows that the size of the penis varies significantly across geographical regions (Figures 5 and 6).

In terms of race and ethnicity of American men, the study by Barboza, et al,^11^ was conducted on Brazilians, ≥18 years old who self-declared as having black or white skin color. The other related study was performed by Sole et al^12^ In this study, the sample was composed of male patients born in Argentina and from four hospitals located in different regions of Argentina (the Department of Urology at Hospital Italiano, Buenos Aires; Sanatorio Allende, Córdoba; Hospital Privado de la Comunidad, Mar del Plata; and Hospital Central, Mendoza). The other American-related studies were from the USA.

### Measurement Method the Penile Size

To determine the penile length, the measurement of the stretched penile was conducted from the pubopenile skin junction to the tip of the glans. Before the patient was given anesthesia, the measurements were obtained in the preoperative holding area. The reliability and reproducibility of this measurement were verified.^11,13^

The length of the flaccid penis was measured from the pubopenile skin junction to the meatus. The stretched penis was measured from the pubopenile skin junction to the meatus with the maximum phallus extension, the depth of the prepubic fat pad from the pubopenile skin junction to the pubic bone, and the penile circumference at the midshaft (in centimeters).^14^

Another study reported the measurement of the length of the flaccid penis as the pubic bone (exerting pressure on the prepubic fat) and the tip of the glans with the foreskin retracted as reference points. The circumference was measured at the base of the flaccid penis. To measure a flaccid stretched penis, the same abovementioned criteria were considered, from pubic bone to glans tip with retracted foreskin, stretching it to the maximum.^12^ Table 2 represents the details of the penile measurement method in the included studies.

## Discussion

Our study shows that the size of the penis varies significantly across geographical regions. We summarized the available data in a visually representative graphic. According to the available data, men living in the Americas have the largest stretched penile size and largest flaccid length and circumference; Western Pacific Asian men seem to have the smallest penis size. Although we found statistically significant differences between different WHO regions regarding penis size and circumference, the importance of the length of the penis as a measure of masculinity and its value in sexuality is limited at best. Nevertheless, due to various issues accentuated in modern times, any minimal difference in penis size and circumference has an impact on the body image and self-perception of men.

The topic of penis size is often a subject of curiosity and discussion among individuals. However, it is essential to approach this topic with sensitivity and respect for diverse perspectives. It is important to recognize that there is no universally agreed-upon “ideal” penis size for women. Preferences vary among individuals, and factors such as emotional connection, communication, and overall sexual compatibility play a significant role in sexual satisfaction. Studies have consistently shown that penis size is not the primary determinant of sexual pleasure for most women. Other factors, such as foreplay, emotional intimacy, and communication, are often more crucial for a fulfilling sexual experience. Focusing solely on penis size oversimplifies the complex nature of human sexuality and reduces it to a physical attribute. It is essential to embrace a holistic understanding of sexual pleasure that encompasses emotional, psychological, and physical aspects. Men who have concerns about their penis size may experience anxiety, self-esteem issues, and feelings of inadequacy. It is crucial to provide support and understanding in such cases. Society often perpetuates unrealistic beauty standards, including those related to genitalia. It is crucial to promote body positivity and self-acceptance for all individuals, regardless of their physical attributes. Encouraging individuals to embrace their bodies and focus on overall well-being rather than conforming to societal expectations fosters a healthier and more positive mindset. Furthermore, educating society about the diversity of human bodies and promoting acceptance and respect for individual differences is vital in creating a more inclusive and compassionate society. Discussions surrounding the ideal penis size for women and concerns related to small penis size need to be approached with sensitivity, respect, and a focus on promoting body positivity. Recognizing the complex nature of human sexuality and fostering open communication and emotional intimacy can contribute to a more fulfilling and satisfying sexual experience for individuals and their partners. Ultimately, it is crucial to shift the focus from physical attributes to overall well-being and acceptance of oneself and others.^2,15^

A serious limitation to our findings is the lack of standardization for measuring penis size in different situations,^4^ which may have led to a systematic bias. Greenstein et al have provided recommendations for the accurate measurement of penis length and girth in clinical research and practice.^4^ Such recommendations could be used in future research to limit the dispersion and heterogeneity in the data. Indeed, we detected considerable heterogeneity and dispersion in penis size within (WHO) regions. For example, the small number of included studies and the differences in their sizes could explain the observed heterogeneity. Veale and colleagues relied on data from 10 704 men to build nomograms as tools for clinicians to have a better understanding of the normality in penis length and circumference. Their meta-analyses for outcomes such as flaccid penis length, stretched penis length, erect penis length, flaccid penis circumference, and erect penis circumference were similar to those of our study; however, our study comprised a larger cumulative sample size.^8^

Data such as the ones reported in this paper are necessary to decondition false assumptions and expectations. For example, on the world data website, the erect penis size in African countries (15-17.6 cm) was much larger than that in the others, still, the erected penile size of Asian and Pacific men was reported as the lowest (<12 cm) (https://www.worlddata.info/average-penissize.php). Unfortunately, there is no data to support these claims making them selective at best. Such misleading data can cause psychological and sociological falsehoods and sociocultural deception. Probably, the differences within a region are larger than that between the regions.

Regarding the erect penis length and circumference, we could not find any statistically significant difference between the regions. This can be due to the small number of enrolled studies and their relatively low sample sizes. Another reason could be that the practical measurement of the erected penis in a clinical setting has some socio-cultural limitations.^9^

### Potential Limitations and Biases

Our study has many limitations. The sample size of the enrolled studies might not be representative of the regional population. On the other hand, we did not have adequate high-quality studies from some regions such as Africa and Southeast Asia. There was very limited data to adjust the penile size for body mass index and body image of the participants in the enrolled studies. Finally, the standard method for measurement of the penile size is still unclear.

Another valid concern of the current study is regarding the intermixing of geography and ethnicity descriptors. For instance, comparing “men living in the Americas” with “Western Pacific Asian men” may lead to confusion, as Asian men originating from the Western Pacific could be included in the Americas WHO location if they are living in the Americas. There is variability in how penile size measurements were conducted across different studies. Although some studies reported standardized methods, others may have used non-standardized or subjective techniques, potentially affecting the reliability and comparability of the data. The findings indicate significant variations in penile size across geographical regions. However, cultural factors influencing body image and health-seeking behavior may affect the populations studied, leading to potential biases in the represented data. While some studies included diverse ethnic groups, others focused on specific populations (e.g., Brazilian or Argentine men). This lack of ethnic diversity may limit the applicability of the findings to other racial or ethnic groups. The age range of participants across studies was not uniformly reported. Variations in age demographics could influence penile size measurements, as size can change with age, potentially confounding results. Furthermore, studies with significant or favorable results are more likely to be published than those with null findings. This publication bias could lead to an overestimation of average penile sizes reported in the literature. Although the risk of bias in included studies was deemed moderate/low, the decision not to exclude any study may mask underlying biases that could affect the overall conclusions. The definitions of “erect,” “flaccid,” and “stretched” penile lengths may vary across studies, leading to inconsistencies in reported measurements and complicating direct comparisons. In addition, the clinical implications of the findings regarding penile size variations were not thoroughly addressed. The results may not translate into meaningful clinical or health-related outcomes for individuals, limiting their practical application.

In light of this issue, further research can better account for these factors and strive for more robust methodologies and inclusive study designs, and encouraged to consider framing the takeaway as follows: “Penis size measurements indicate larger sizes within the described locations” rather than implying that penis sizes are inherently larger in these areas. This revised approach aligns with the limitations of the data presented in the current study and appears to be a more appropriate interpretation.

## Conclusions

Penis size varies significantly across WHO regions, requiring region-adjusted standards as a basis for patient and partner counseling. These data provide a framework for discussion of body image expectations and therapeutic strategies in this sensitive personal and subjective topic. A high-quality multicenteric study with a standard measurement of penile size in different regions could help provide more reliable evidence to support our unexpected findings.

## Figures and Tables

**Table 1.** Quality Assessment of the Included Studies Using the JBI Critical Appraisal Checklist for Cross-Sectional Studies.

Author/Year	Q1	Q2	Q3	Q4	Q5	Q6	Q7	Q8	Q9
Takure, 2021	Yes	Yes	Unclear	Yes	Yes	Yes	Unclear	Yes	NA
Mauro, 2021	Yes	Yes	Yes	Yes	Yes	Yes	Yes	Yes	NA
Hoai, 2021	Yes	Yes	Yes	Yes	Yes	Yes	Yes	Yes	NA
Solé, 2022	Yes	Yes	Unclear	Yes	Yes	Yes	Yes	Yes	NA
Chrouser, 2013	Yes	Yes	Unclear	Yes	Yes	Yes	Unclear	Yes	NA
S,engezer, 2002	Yes	Yes	Unclear	Yes	Yes	Yes	Yes	Yes	NA
Ajmani, 2015	Yes	Yes	Unclear	Yes	Yes	Yes	Yes	Yes	NA
Ponchietti, 2001	Yes	Yes	Yes	Yes	Yes	Yes	Yes	Yes	NA
Aslan, 2011	Yes	Yes	Yes	Yes	Yes	Yes	Yes	Yes	NA
Schneider, 2001	Yes	Yes	Unclear	Yes	Yes	Yes	Yes	Yes	NA
Habous, 2015	Yes	Yes	Yes	Yes	Yes	Yes	Yes	Yes	NA
Chen, 2014	Yes	Yes	Yes	Yes	Yes	Yes	Yes	Yes	NA
Wessells, 1996	Yes	Yes	Unclear	Yes	Yes	Yes	Yes	Yes	NA
Chen, 2000	Yes	Yes	Unclear	Yes	Yes	Yes	Yes	Yes	NA
Spyropoulos, 2002	Yes	Yes	Yes	Yes	Yes	Yes	Yes	Yes	NA
Son, 2003	Yes	Yes	Unclear	Yes	Yes	Yes	Unclear	Yes	NA
Awwad, 2005	Yes	Yes	Unclear	Yes	Yes	Yes	Yes	Yes	NA
Mehraban, 2007	Yes	Yes	Yes	Yes	Yes	Yes	Yes	Yes	NA
Promodu, 2007	Yes	Yes	Unclear	Yes	Yes	Yes	Yes	Yes	NA
Khan, 2012	Yes	Yes	Yes	Yes	Yes	Yes	Yes	Yes	NA
Söylemez, 2012	Yes	Yes	Yes	Yes	Yes	Yes	Yes	Yes	NA
Herbenick, 2013	Yes	Yes	Yes	Yes	Yes	Yes	Unclear	Yes	NA
Savoie, 2003	Yes	Yes	Unclear	Yes	Yes	Yes	Unclear	Yes	NA
Siminoski, 1993	Unclear	Unclear	Unclear	Unclear	Yes	Unclear	Unclear	Yes	NA
Barboza, 2017	Yes	Yes	Yes	Yes	Yes	Yes	Yes	Yes	NA
Shalaby, 2014	Yes	Yes	Yes	Yes	Yes	Yes	Yes	Yes	NA
Yeung, 2017	Unclear	Unclear	Unclear	Unclear	Unclear	Unclear	Unclear	Unclear	Unclear
Choi, 1999	Yes	Yes	Unclear	Yes	Yes	Yes	Unclear	Yes	NA
Tomova, 2010	Yes	Yes	Yes	Yes	Yes	Yes	Yes	Yes	NA
Bondil, 1992	Yes	Yes	Unclear	Yes	Yes	Yes	Unclear	Yes	NA
Kamel, 2009	Yes	Yes	Yes	Yes	Yes	Yes	Yes	Yes	NA
Suzuki, 2019	Yes	Yes	Unclear	Yes	Yes	Yes	Yes	Yes	NA
Gooran, 2016	Yes	Yes	Unclear	Yes	Yes	Yes	Unclear	Yes	NA

Q1. Was the sample frame appropriate to address the target population?

Q2. Were study participants sampled in an appropriate way?

Q3. Was the sample size adequate?

Q4. Were the study subjects and the setting described in detail?

Q5. Was the data analysis conducted with sufficient coverage of the identified sample?

Q6. Were valid methods used for the identification of the condition?

Q7. Was the condition measured in a standard, reliable way for all participants?

Q8. Was there an appropriate statistical analysis?

Q9. Was the response rate adequate, and if not, was the low response rate managed appropriately?

**Table 2.** Measurement Method the Penile Size

Author/Year	Measurement
Takure, 2021	The flaccid penile length (FPL) and stretched penile length (SPL) were prospectively measured in centimeters from the pubic arch to the tip of the glans penis.
Mauro, 2021	All measurements were performed under similar environmental conditions (air-conditioned room and at temperatures varying from 23°C to 25°C). Penile length was measured along the dorsum of the penis by a ruler with millimeter markings, with the patients standing up. The penile dimensions assessed were penile length from the pubopenile skin vertex, depressing the pubic fat, to the extremity of the glans, with the ruler placed against the dorsal part of the penis and the circumference, the diameter at the midpoint of the penile shaft, in flaccidity and in erection.
Hoai, 2021	Penile dimensions were taken once by 5 well-trained andrologists following a consistent measurement protocol immediately after penile exposure to minimize temperature effects. Penile parameters were measured by a rigid ruler for lengths and a steel caliper for diameters in the standing position with the penis held parallel to the floor. Penile length was the linear distance from the pubopenile junction to the tip of the glans along the dorsal side by pushing the pre-pubic fat pad to the bone in the flaccid and fully stretched state without an erection. Penile diameters were defined as the line between two points on the circumference of the penile shaft’s middle point, and the corona of the glans that concludes their center.
Solé, 2022	To measure the length of the flaccid penis, the pubic bone (exerting pressure on the prepubic fat) and the tip of the glans with the foreskin retracted were taken as reference points. The circumference was measured at the base of the flaccid penis. To measure a flaccid stretched penis, the same abovementioned criteria were considered, from pubic bone to glans tip with retracted foreskin, stretching it to the maximum
Chrouser, 2013	Penile measurements were made immediately before surgery after administering local anesthesia. After the manual reduction of any suprapubic fat, gentle traction was applied at the corona. Stretched penile length in cm was measured from the penopubic junction to the most distal glans using a ruler. Glans length in cm from corona to distal glans was also recorded. Length in cm from the corona to the distal edge of the reduced foreskin in the relaxed state was measured using a ruler. Unstretched shaft circumference in cm at the corona and distal shaft was measured with a tape measure. The distal foreskin stretched diameter was measured in cm by inserting the inside jaws of a vernier caliper in the opening of the reduced foreskin and gently separating until the skin was taut transversely, ensuring that the foreskin did not roll off. Foreskin thickness under tension in mm was measured by placing the fixed jaw of the caliper flush along the longitudinal penile axis with the tip at the corona and then reducing the foreskin over the glans and the caliper jaw. The jaws were then approximated until the foreskin did not slide out with care taken not to over compress the tissue while measuring
S Engezer, 2002	Measurements were made immediately after the patient was undressed to minimize the effect of temperature. Tape measurements of the penis were obtained to the nearest 0.5 cm by the same examiner. All measurements were acquired at room temperature. The flaccid length of the penis was measured as the distance between the peno-pubic skin junction and the tip of the glans in the nonerected and nonstretched state of the penis. The point of the penopubic junction was determined by the crease that was formed by gentle handling of the penis dorsally. The erect penile length was measured at full erection without the use of any pharmacological agents after a period of privacy and self-stimulation. The stretched penile length was determined from the penopubic junction to the tip of the glans penis by placing the end of a straight-edge ruler gently against the pubic ramus and applying traction along the length of the phallus to the point of increased resistance—an easily appreciated endpoint.
Ajmani, 2015	Total length of the penis (flaccid position) was measured from the root of the penis to the tip of the glans on its dorsal surface by sliding caliper. The circumference of the penis was measured in the middle part of the shaft of the penis by measuring tape. Circumference of the scrotum was measured from the right scrotal base along its circumference to the left scrotal base by measuring tape. Length of the testis was measured between the two most distant points of its longitudinal axis by sliding caliper. Width of the testis was measured in the mid portion of the testis by sliding caliper. Measurements 4 and 5 were done on both the left and right testis.
Aslan, 2011	Penile measurements were performed between 10:00 a.m. and 4:00 p.m. under ambient light and room temperature with subjects standing up and with the penis held parallel to the floor. Penile length was measured by a ruler with millimeter markings along the dorsum of the penis from the pubopenile junction to the tip of the glans (meatus) while under maximal, but not painful, extension. The measurement was performed during both flaccid and stretched states. The testicular volume of the subjects was measured by bimanual palpation using a Prader orchidometer with volumes ranging from 1 to 25 cm^3^ (1-6, 8, 10, 12, 15, 20, and 25 cm^3^) after simultaneously stretching the scrotal skin over the testis. Testicular volumes between 15 and 25 cm^3^ were considered within the normal range.
Schneider, 2001	All measurements were performed in the same room at the same temperature. The penile length measured was done in the flaccid state from the pubic-penile skin junction to the meatus with a ruler. The prepubic fat pad was pushed to the bone at the maximum. The penile width was measured at the base and the glans penis with a caliper. Patients were left for a period in privacy and after self-stimulation (visual and manual), all measurements were repeated at full erection.
Habous, 2015	Each patient had three parameters of the erect penis recorded: circumference of the penile shaft; penile length from the suprapubic skin to distal glans (skin to tip); and penile length pubis to distal glans (bone to tip). Using a rigid plastic ruler, skin-to-tip measurement was conducted as follows: with the penis in full erection, the base of the ruler was placed on the peno-pubic skin junction and the tip of the ruler was placed at the level of the tip of the glans penis. Bone-to-tip measurement was conducted as above except the base of the ruler was pushed firmly down to the pubic bone. The penile circumference was measured with a tape at the base of the penis. Weight, height, BMI and age were also recorded.
Chen, 2014	The participants were kept in the supine position. Penile length was measured by a straight-edge ruler, and penile circumference and glans dimensions were measured using a vernier caliper. The flaccid penile length was measured as the linear distance along the dorsal side of the pubopenile skin junction to the tip of the glans. The stretched flaccid length was measured as the distance from the pubic bone to the tip of the glans, under gentle painless extension of the penis. The erect length was measured as the distance from the pubic bone to the tip of the glans. The penile circumference was measured at the middle of the shaft. The glans length was measured as the distance from the corona to the tip of the glans, whereas the glans diameter was measured as the linear distance between two points on the circumference of the glans that passes through its center.
Herbenick, 2013	from the centimeter-based measure of their erect penile dimensions (erect length and circumference.
Promodu, 2007	Penile length was defined as the linear distance along the dorsal side of the penis extending from the pubopenile skin junction to the tip of the glans. Penile circumference was measured at the middle of the shaft.
Awwad, 2005	Penile measurements were taken immediately after the patient undressed to minimize the effect of temperature and touch on penile size. The measurements were taken while the patients were lying down and the legs slightly abducted. The flaccid and fully stretched penile lengths were measured in both groups. In addition, midshaft circumference in the flaccid state was measured in group one, and in the second group, penile length in the fully erect penis was measured. Full erection state was achieved using intracavernosal injection of trimex (each 1 ml of trimex is composed of papaverine 30 mg, phentolamine 1 mg, and prostaglandin 10 mg). The dose of trimex ranged from 0.1 to 0.4 ml. Patients who did not achieve full nonbendable erection were excluded from the study. A measuring tape was used to measure the length and the midshaft circumference of the penis. The starting point was on the dorsal aspect of the penis at its base at the pubic–penile skin junction, pushing the prepubic fat pad against the pubic bone while the tip of the penis was the other reference point. Measurements were taken by the two urologists, and endocrinologists involved in the investigation for groups one and two, and each one was instructed on the same and exact method of measurement. In addition, several measurements were repeated by two investigators on different visits and found to be similar. All measurements were approximated to the nearest 0.5 cm.
Mehraban, 2007	Tape measurement of penis, glans and penile circumference was made in centimeters with one decimal point. To measure the total penile length, we placed the butt of a rigid tape measure tool on the pubic skin over the dorsum of the fully starched penis held by the other hand and stretched once. The distance between the pubic skin and the external urethral meatus was recorded. The glans length was measured from the corona to the external urethral meatus level. The penile circumference or girth was measured at the midshaft. The height and weight were measured as routine.
Wessells, 1996	Tape measurements of the flaccid and erect penis were obtained to the nearest 0.5 cm. by 1 examiner. Flaccid length, circumference, depth of the prepubic fat pad and stretched penile length were measured immediately after the patient undressed to minimize the effects of temperature. Measurements were made of the length from the pubopenile skin junction to the meatus, circumference at the mid-shaft and fat pad depth by pushing the tape into the pubic bone. Stretched flaccid length was measured from the pubopenile skin junction to the meatus under maximal extension of the phallus. All patients underwent intracavernous injection of prostaglandin El for evaluation of erectile dysfunction. After a period of privacy and self-stimulation, penile length and circumference were measured at full erection.
Chen, 2000	All penile measurements and evaluations were performed by the same investigator, thereby eliminating inter-observer variations. Only patients who achieved full erection (and without penile curvature) were included in the analysis. The study was conducted in a dimly lit private room, and the patient was in supine position. Penile length was measured by a caliper. While measuring penile length at the dorsal aspect, the caliper was pushed into the pubic bone in order to eliminate the effect of the pubic fat pad. Penile length was measured dorsally from the pubo-penile angle to the meatus, and ventrally from the penoscrotal junction to the meatus side. Circumference was measured by an ordinary ‘tumo-meter’ at the penile base and at the corona. Penile dimensions were measured in a flaccid state and during axial stretching in which the glans was gently stretched until the patient expressed discomfort.
Söylemez, 2012	Penile length was measured along the dorsum of the penis by a ruler with millimeter markings, with the subjects standing up. The penile measurements assessed were the circumference; the diameter at the midpoint of the penile shaft, at the flaccid length; from the pubopenile skin vertex, depressing the pubic fat, to the extremity of the glans, with the ruler placed against the dorsal part of the penis, at the fully-stretched length; and from the pubopenile skin vertex, under maximal—but not painful—extension and depressing the pubic fat, to the extremity of the glans with the ruler placed against the dorsal part of the penis.
Spyropoulos, 2002	All measurements were performed by the same examiner under similar environmental conditions (room light and temperature) and included: 1. Ultrasonographic testicular volume calculation using the formula with the transducer operating in the frequency of 7.5 MHz. 2. Tape measurements of the flaccid, slightly stretched, penile shaft with the subject supine and his legs flat-adducted of the (a) length from the pubopenile skin junction (from the skin over the pubis) to the base of the glans (coronal ridge) in the dorsal surface of the penis (Lshaft), under maximal, but painless, extension of the phallus, (b) circumference at the base of the penis (p1), and (c) circumference at the base of the glans (coronal groove; p2). 3. Measurements of the flaccid glans penis length, dorsally from the coronal ridge to the tip of the glans.
Son, 2003	The flaccid penile length, flaccid mid-shaft circumference, stretched length and pre-pubic bone fat pad depth were measured in a warm comfortable environment. The accuracy the subjects assessed their penile size was investigated by asking them to rate their penile size, as “very small,” “small,” “normal,” “large” or “very large,” All subjects were asked to complete the Minnesota multiphasic personality inventory (MMPI) test.
Khan, 2012	1. The flaccid ‘pendulous’ length, in which the flaccid penile length was measured from the tip of the glans penis to the base of the pendulous penis. 2. The ‘pubic arch penile length’, measuring from the tip of the glans penis to the lower edge of the pubic bone above the suspensory ligament insertion. 3. The ‘stretched flaccid length’, which is determined as the distance from the pubic bone to the tip of the glans penis under a gentle painless extension of the penis. Penile length measurements were taken at normal room temperature with the patient supine and the legs adducted and before surgery in anaesthetized patients. Only Caucasian, British men were included in the study. Additionally, testicular size was measured using an orchidometer. The patient’s age and the reason for referral were also recorded.
Savoie, 2003	The penile measurements (cm.) consisted of flaccid penis length from the pubopenile skin junction to the meatus, stretched penis length from the pubopenile skin junction to the meatus with maximal extension of the phallus, depth of prepubic fat pad from the pubopenile skin junction to the pubic bone and penile circumference at midshaft.
Siminoski, 1993	Stretched penile length (Schonfeld, 1943; Schonfeld & Beebe, 1942).
Barboza, 2017	The researcher measured the penis length in real fully-stretched flaccid length (RSLmax), from the pubopenile junction, depressing the pubic fat until the pubis bone, to the tip of the glans with the ruler placed against the dorsal surface of the penis, with the penis in its maximal extension.
Shalaby, 2014	1 Measurement of penile length using a tape while the patient standing and the penis held parallel to the floor, and the penis is stretched as comfortably as possible. Two Measurements of the glans penis length from the corona to the external urethral meatus.
Yeung, 2017	
Choi, 1999	Measurement tools (paper tapes) were designed for user convenience and to minimize measurement errors. Respondents were carefully instructed on how to use the tapes for measuring their penile size after self-stimulation. The distances between marks on the returned tapes were measured with a steel ruler to the nearest 1 mm.
Tomova, 2010	The stretched penile length in the flaccid state was measured with a rigid tape from the pubopenile skin junction to the top of the penis, excluding the prepuce under maximal but not painful extension. The penile circumference was measured at the base of the penis (close to the pubis) with a measuring tape. For obese males, the abdominal adipose tissue was shifted manually to one side to measure penile length and circumference.
Bondil, 1992	Three penile lengths were measured: FI = initial length, Fmax = maximum stretching length and F2 = after-stretching length. The stretching ability (S) was calculated as S = Fmax - FI, and the elasticity ability (E) asE = F2-Fl.
Kamel, 2009	Measurement of the penile length and girth: as described by Wessels et al. [3]—in the fully stretched state using a tape measure and rigid ruler to confirm measurements, with firm pressure on the pubic bone in a recumbent position. Firm pressure at the pubic bone using a rigid ruler attempts to reduce measurement bias related to being overweight. The measurements were performed instantly after penile exposure to diminish the effects of temperature. The stretched penile length was defined as the linear distance between the symphysis pubis to the tip of the glans in the fully stretched state (still flaccid). The penile girth was estimated at the mid penile shaft.
Suzuki, 2019	Penile size was measured by the method Wessells et al reported.4 Briefly, PFL was measured by a ruler along the dorsum of the penis from the pubopenile junction to the tip of the glans. PSL was measured under the maximal extension of the phallus to estimate erect penile length. Penile circumference was measured at the mid-shaft of the penis. All the measurements of penile size were carried out by the same examiner at room temperature with the cadaver in the supine position on the table.
Gooran, 2016	The penis length was measured during the erection period at the dorsal side and while it was completely pulled. The penis length was measured from under the symphysis of the pubis to the meatus, and the length of mucosa was spanned from the corona to the place mucosa–skin junction on the ventral side.

**Table:** 

Search Number	Query	Sort By	Filters	Search Details	Results
26	((((((((organ* size*[Text Word]) OR (organ* volume*[Text Word])) OR (organ* girth*[Text Word])) OR (organ* measurement*[Text Word])) OR (organ* length[Text Word])) OR (organ* circumference[Text Word])) OR (organ* dimension*[Text Word])) OR (“Organ Size”[Mesh])) AND (((“Penis”[Mesh]) OR “Penile Erection”[Mesh]) OR (((((phallus[Text Word]) OR (penis[Text Word])) OR (penile[Text Word])) OR (genital*[Text Word])) OR (peni[Text Word])))			(“organ size*”[Text Word] OR “organ volume*”[Text Word] OR (“organ*”[All Fields] AND “girth*”[Text Word]) OR “organ measurement*”[Text Word] OR (“organ*”[All Fields] AND “length”[Text Word]) OR (“organ*”[All Fields] AND “circumference”[Text Word]) OR “organ dimension*”[Text Word] OR “Organ Size”[MeSH Terms]) AND (“Penis”[MeSH Terms] OR “Penile Erection”[MeSH Terms] OR (“phallus”[Text Word] OR “Penis”[Text Word] OR “penile”[Text Word] OR “genital*”[Text Word] OR “peni”[Text Word]))	3015
24	(((“Body Weights and Measures”[Mesh]) OR (“Organ Size”[Mesh])) OR (((((((organ* size*[Text Word]) OR (organ* volume*[Text Word])) OR (organ* girth*[Text Word])) OR (organ* measurement*[Text Word])) OR (organ* length[Text Word])) OR (organ* circumference[Text Word])) OR (organ* dimension*[Text Word]))) AND (((“Penis”[Mesh]) OR “Penile Erection”[Mesh]) OR (((((phallus[Text Word]) OR (penis[Text Word])) OR (penile[Text Word])) OR (genital*[Text Word])) OR (peni[Text Word])))			(“Body Weights and Measures”[MeSH Terms] OR “Organ Size”[MeSH Terms] OR (“organ size*”[Text Word] OR “organ volume*”[Text Word] OR (“organ*”[All Fields] AND “girth*”[Text Word]) OR “organ measurement*”[Text Word] OR (“organ*”[All Fields] AND “length”[Text Word]) OR (“organ*”[All Fields] AND “circumference”[Text Word]) OR “organ dimension*”[Text Word])) AND (“Penis”[MeSH Terms] OR “Penile Erection”[MeSH Terms] OR (“phallus”[Text Word] OR “Penis”[Text Word] OR “penile”[Text Word] OR “genital*”[Text Word] OR “peni”[Text Word]))	5090
25	(((((((organ* size*[Text Word]) OR (organ* volume*[Text Word])) OR (organ* girth*[Text Word])) OR (organ* measurement*[Text Word])) OR (organ* length[Text Word])) OR (organ* circumference[Text Word])) OR (organ* dimension*[Text Word])) OR (“Organ Size”[Mesh])			“organ size*”[Text Word] OR “organ volume*”[Text Word] OR (“organ*”[All Fields] AND “girth*”[Text Word]) OR “organ measurement*”[Text Word] OR (“organ*”[All Fields] AND “length”[Text Word]) OR (“organ*”[All Fields] AND “circumference”[Text Word]) OR “organ dimension*”[Text Word] OR “Organ Size”[MeSH Terms]	168 330
23	((“Body Weights and Measures”[Mesh]) OR (“Organ Size”[Mesh])) OR (((((((organ* size*[Text Word]) OR (organ* volume*[Text Word])) OR (organ* girth*[Text Word])) OR (organ* measurement*[Text Word])) OR (organ* length[Text Word])) OR (organ* circumference[Text Word])) OR (organ* dimension*[Text Word]))			“Body Weights and Measures”[MeSH Terms] OR “Organ Size”[MeSH Terms] OR (“organ size*”[Text Word] OR “organ volume*”[Text Word] OR (“organ*”[All Fields] AND “girth*”[Text Word]) OR “organ measurement*”[Text Word] OR (“organ*”[All Fields] AND “length”[Text Word]) OR (“organ*”[All Fields] AND “circumference”[Text Word]) OR “organ dimension*”[Text Word])	692 422
22	(“Body Weights and Measures”[Mesh]) OR (“Organ Size”[Mesh])			“Body Weights and Measures”[MeSH Terms] OR “Organ Size”[MeSH Terms]	620 121
21	“Body Weights and Measures”[Mesh]	Most Recent		“Body Weights and Measures”[MeSH Terms]	620 121
18	((((((organ* size*[Text Word]) OR (organ* volume*[Text Word])) OR (organ* girth*[Text Word])) OR (organ* measurement*[Text Word])) OR (organ* length[Text Word])) OR (organ* circumference[Text Word])) OR (organ* dimension*[Text Word])			“organ size*”[Text Word] OR “organ volume*”[Text Word] OR (“organ*”[All Fields] AND “girth*”[Text Word]) OR “organ measurement*”[Text Word] OR (“organ*”[All Fields] AND “length”[Text Word]) OR (“organ*”[All Fields] AND “circumference”[Text Word]) OR “organ dimension*”[Text Word]	166 989
5	“Organ Size”[Mesh]	Most Recent		“Organ Size”[MeSH Terms]	93 335
4	((“Penis”[Mesh]) OR “Penile Erection”[Mesh]) OR (((((phallus[Text Word]) OR (penis[Text Word])) OR (penile[Text Word])) OR (genital*[Text Word])) OR (peni[Text Word]))			“Penis”[MeSH Terms] OR “Penile Erection”[MeSH Terms] OR “phallus”[Text Word] OR “Penis”[Text Word] OR “penile”[Text Word] OR “genital*”[Text Word] OR “peni”[Text Word]	179 808
3	((((phallus[Text Word]) OR (penis[Text Word])) OR (penile[Text Word])) OR (genital*[Text Word])) OR (peni[Text Word])			“phallus”[Text Word] OR “penis”[Text Word] OR “penile”[Text Word] OR “genital*”[Text Word] OR “peni”[Text Word]	158 546
1	(“Penis”[Mesh]) OR “Penile Erection”[Mesh]	Most Recent		“Penis”[MeSH Terms] OR “Penile Erection”[MeSH Terms]	46 426

## Data Availability

The authors confirm that the data supporting the findings of this study are available within the article [and/or] its supplementary materials.

## Appendix 1. The Search Strategy of PubMed.

**Cochrane**

(([mh Penis]) OR (Penis:ti,ab,kw OR penile:ti,ab,kw OR phallus:ti,ab,kw)) AND (([mh “Organ Size”]) OR (Size:ti,ab,kw OR girth:ti,ab,kw OR measurement:ti,ab,kw OR length:ti,ab,kw OR circumference:ti,ab,kw OR dimension*:ti,ab,kw))

**Embase**

((Penis/exp) OR (Penis OR penile OR phallus)) AND ((‘Organ Size’/exp) OR (Size OR girth OR measurement OR length OR circumference OR dimension*))

**Scopus**

((INDEXTERMS(Penis)) OR (TITLE-ABS-KEY(Penis) OR TITLE-ABS-KEY(penile) OR TITLE-ABS-KEY(phallus))) AND ((INDEXTERMS(“Organ Size”)) OR (TITLE-ABS-KEY(Size) OR TITLE-ABS-KEY(girth) OR TITLE-ABS-KEY(measurement) OR TITLE-ABS-KEY(length) OR TITLE-ABS-KEY(circumference) OR TITLE-ABS-KEY(dimension*)))
